# Accessible ethics and legal advice for wastewater surveillance: The WWS ethics adviser app

**DOI:** 10.1371/journal.pwat.0000422

**Published:** 2026-01-30

**Authors:** Tsaone Tamuhla, Mercury Shitindo, Michelle Nichols, Vincent Junxiong Pang, Elyssa Jiawen Liu, Nicki Tiffin

**Affiliations:** 1 South African National Bioinformatics Institute, University of the Western Cape, Cape Town, South Africa; 2 Africa Bioethics Network, Nairobi, Kenya; 3 University of Zaragoza, Zaragoza, Spain; 4 Department of Health Sciences and Research, Medical University of South Carolina, College of Health Professions, Charleston, South Carolina, United States of America; 5 Centre for Outbreak Preparedness, Duke-NUS Medical School, Singapore, Singapore; 6 SingHealth Duke-NUS Global Health Institute, Duke-NUS Medical School, Singapore, Singapore

## Abstract

Wastewater surveillance (WWS) has a long history in infectious disease management, from the detection of *Salmonella Paratyphi B* in sewers by Moore in 1948, to its role in global poliovirus eradication and more recently the deployment of WWS to track SARS-CoV-2. Whilst current interest has focused on pathogen sequencing to monitor outbreaks, WWS encompasses a much broader molecular landscape that includes a wide array of chemical compounds, proteins and DNA from human, animal, plant and microbial organisms. This diversity creates novel opportunities for public health applications, environmental monitoring, biodiversity studies, forensic science and even commercial innovation. The communities from which wastewater is sourced should be the primary beneficiaries of surveillance efforts, but WWS also requires robust governance mechanisms and ethical oversight to prevent harm to communities, ensure equitable practices, fulfil legal obligations and ensure appropriate use of potentially sensitive or commercially valuable findings. End-users of WWS resources must navigate these complexities to ensure ethical and legal compliance and responsible use of WWS samples and data. To assist with this process, we developed the WWS Ethics Adviser, an online interactive tool designed to alert users to context-specific ethical, legal, and governance considerations in WWS activities. The app aims to synthesise the principles of guidelines, treaties and other complex frameworks and resources into concise and actionable information for end users. The content is created within a two-dimensional matrix that categorises molecular entities and their origin against corresponding ethical, legal and governance elements. The tool offers tailored advice for WWS practitioners, oversight committees and responsible parties, in order to support equitable, ethical and legal WWS practices across diverse settings and use cases, and can be found at: https://wastewater-surveil-lance-ethics.streamlit.app/.

## Introduction

Wastewater surveillance (WWS) has a long history in public health infectious disease management: in the United Kingdom in 1948 the “Moore swab” was used as an environmental surveillance tool, suspended as a gauze filter at various points in the sewer system to capture and trace *Salmonella Paratyphi B* in sewers to pinpoint the residence of a chronic carrier responsible for sporadic outbreaks of paratyphoid fever [1]. Subsequently, wastewater surveillance (WWS) has also been harnessed in the global effort to eradicate poliovirus [2], and the Global Polio Eradication Initiative used sequencing of environmental sewage to complement other ongoing efforts to identify polio cases [3,4]. During the recent SARS-CoV-2 pandemic, WWS became a core component of monitoring general pathogen disease burden [5,6] using targeted sequencing to amplify and analyse pathogen-specific nucleic acids in collected samples. Since then, WWS has gained increasing visibility as a sustainable and informative approach for monitoring pathogens in the general population, including early detection of outbreaks, new pathogens and new variants of known pathogens.

Whilst the current focus is on pathogen sequencing in wastewater samples [7], it is important to remember that wastewater surveillance can also detect DNA from other species, as well as a range of other molecular targets—each contributing its own narrative about the organisms and chemicals present in that specific wastewater environment [8]. These approaches are also used to assay a wide variety of molecules, for example well-established wastewater-based epidemiology has been used to measure synthetic opioids, opiates, amphetamines, cannabinoids and cocaine [9–11], glucocorticoids as a measure of community stress [12] and for forensic sampling of an array of drugs used legally and illegally by specific communities or at specific events [13].

### Stakeholders and their interests in WWS

Key stakeholders and sectors invested in WWS are very diverse. At the heart of all WWS efforts are the communities and populations who should benefit most directly from its outcomes. Within the human health sector, stakeholders include public health laboratories, academic researchers, wastewater and sanitation engineers and technicians, government health service providers, ministries of health, epidemiologists and other policy- and decision-makers.

Beyond health, stakeholders extend to environmental monitoring bodies, biodiversity researchers, agricultural, veterinary and wildlife health agencies, and organisations involved in One Health and antimicrobial resistance (AMR) stewardship—all of whom have vested interests in the sampling and analysis of wastewater. In the private sector, biotechnology companies stand to benefit from novel biological insights generated through the analysis of wastewater samples. These include opportunities for biomarker discovery and the development of diagnostics and therapeutics for infectious diseases. In addition, WWS may reveal previously uncharacterised microbial products and metabolites with potential for commercial exploitation. The possibility of sequencing plant and animal DNA from wastewater may also lead to new discoveries with commercial potential, including applications in agriculture, environmental biotechnology and natural product development.

These diverse stakeholders who are actively engaged in the collection and use of WWS samples and data typically focus on specific molecular targets or species of particular relevance to their own mandate. WWS involves the generation, integration, harmonisation, and interpretation of diverse data originating from multiple sources, agencies and systems, and to ensure comparability and utility, these data must be handled using consistent methodologies, standard operating procedures, and agreed-upon data standards. In doing such data-driven work, however, responsible parties may overlook the broader potential applications of the material they are working with—applications that span a wide variety of organisms, molecules, and disciplines. These broader uses come with an equally wide range of ethical, legal, and equity-related considerations associated with WWS samples and data [14], all of which must be carefully addressed within a deliberate and transparent governance framework.

### Ethical, governance, and legal considerations in wastewater surveillance

Wastewater samples are rich in molecular diversity, containing not only human pathogens but also genetic material from plants, animals, and microorganisms, as well as chemical residues, pharmaceuticals, and illicit substances [15]. Each of these molecular species may raise different questions about data ownership, appropriate use and potential consequences, creating a complex and often confusing landscape for researchers, public health laboratories and ethics review boards alike. With this in mind, custodians of WWS samples and data have an ethical and legal responsibility to consider the full spectrum of possible uses and to manage associated risks appropriately - yet many individuals involved in the day-to-day work are either unaware of these broader ethical and legal dimensions or lack the guidance needed to apply them effectively in practice [16]. These custodians include, for example, laboratory personnel, researchers, data managers, data analysts and government agencies. Institutional ethics review committees may provide oversight prior to sample collection and analysis, but these bodies also often face similar challenges in identifying and addressing the full range of potential use cases, risks and benefits associated with WWS. In addition, there are many different guidance documents, frameworks, treaties, legislation and published principles relating to the different types of data and specimens found in waste water. Increasingly, guidelines and frameworks are being developed for ethics, equity and governance for WWS, but this information can be dense, complicated and also dispersed across different ethics and legal documents.

To address this gap, we have developed a browser-based tool to support responsible custodianship and the onward use of WWS samples and data. The tool is designed to facilitate a clearer understanding of the ethical, legal, equity and governance considerations that should inform work in this domain, helping both practitioners and reviewers navigate the complexity of WWS with greater confidence and accountability. The tool aims to consolidate and communicate in simple language the key principles and concerns, in a single accessible resource that can guide stakeholders about key considerations that they may need to keep in mind.

*Ethical considerations* in WWS extend beyond conventional biomedical research concerns. While there are clear potential benefits—such as early detection of disease outbreaks, improved public health responses and novel scientific insights—there are also risks of harm. These risks are exacerbated where there is a risk of unverified findings or inaccurate analyses and reporting being presented to the public. These may include reputational damage for a community, region or nation, discrimination and stigma for communities, misinterpretation of findings, and the secondary use of data and samples for surveillance beyond the original public health mandate. For example, where sampling location is provided at a building or block level, such as a nursing home or office block, there is a substantial risk of this small, identifiable community being linked to an aggregated infectious disease profile [17]. Ethical principles such as autonomy and respect for persons underscore the need for meaningful community engagement and, where appropriate, mechanisms for community-level informed consent for surveillance activities. Issues of justice and equity demand that those who primarily bear the risks of surveillance should also receive equitable benefits, and particular care must also be taken to avoid reinforcing existing inequalities through biased sampling practices and exclusion of underrepresented groups including those who live outside standard wastewater ecosystems and sampling sites. Informal settlements in highly populated cities and very rural communities, for example, may not have established sanitation infrastructure and will not be represented in WWS that relies on sampling from sewage and water treatment systems [18].

*Governance considerations* relate to the systems, structures, and accountability mechanisms that oversee how WWS samples and data are managed. These include questions about who holds responsibility for sample and data stewardship, what protections are required to ensure confidentiality and data security, and how access to samples and derived data should be governed. A responsible access model should be transparent, inclusive, and proportionate to the risks and sensitivities involved. Transparent and robust governance frameworks, developed and implemented in collaboration with stakeholders, can foster public confidence that wastewater surveillance is being conducted with appropriate safeguards in place.

*Legal considerations* span multiple domains and may vary across jurisdictions. Relevant legislation may include privacy and data protection laws, public and environmental health acts, and regulations governing agricultural and conservation practices. Issues of data sovereignty—especially where WWS captures information from minority or marginalised populations—must be carefully addressed: the Convention on Biological Diversity (CBD, [19]) established in 1992, affirms sovereign rights over genetic resources, species and ecosystem diversity; and the Nagoya Protocol, adopted in 2010, enforces the principle of equitable benefit-sharing from the use of biological resources. In addition, the World Health Organisation (WHO) Pandemic Agreement, adopted in May 2025, outlines next steps towards establishing a multilateral Pathogen Access and Benefit Sharing (PABS) system to regulate access to biological materials and their derived data [20,21]. Even within national borders, questions of intellectual property ownership and fair licensing must also be considered, as WWS may lead to commercially valuable discoveries—such as novel enzymes or active molecules for biotech and pharmaceutical development—with significant economic and public health implications. The legislative requirements are likely to differ across countries, and for this reason the legal considerations are presented in general terms that advise users to consider certain types of legislation that are common to many countries, or are global treaties or agreements, rather than prescribing exact statutes or laws.

## Methods

We created a matrix of two dimensions. The first dimension reflects the molecular entities that may be found in wastewater, including a subtype of the molecular entity where relevant, and also the plant, animal, microbial or environmental origin of the material (Table 1). The second dimension of the matrix deconstructs the ethical and legal considerations, grouping them into the categories of ethics, justice, governance and legislation (Table 2). For each cell of the matrix, we captured the relevant type of ethical/legal considerations for that particular molecule type and subtype from that particular species or environmental origin (S1 Table).

**Fig 1.**
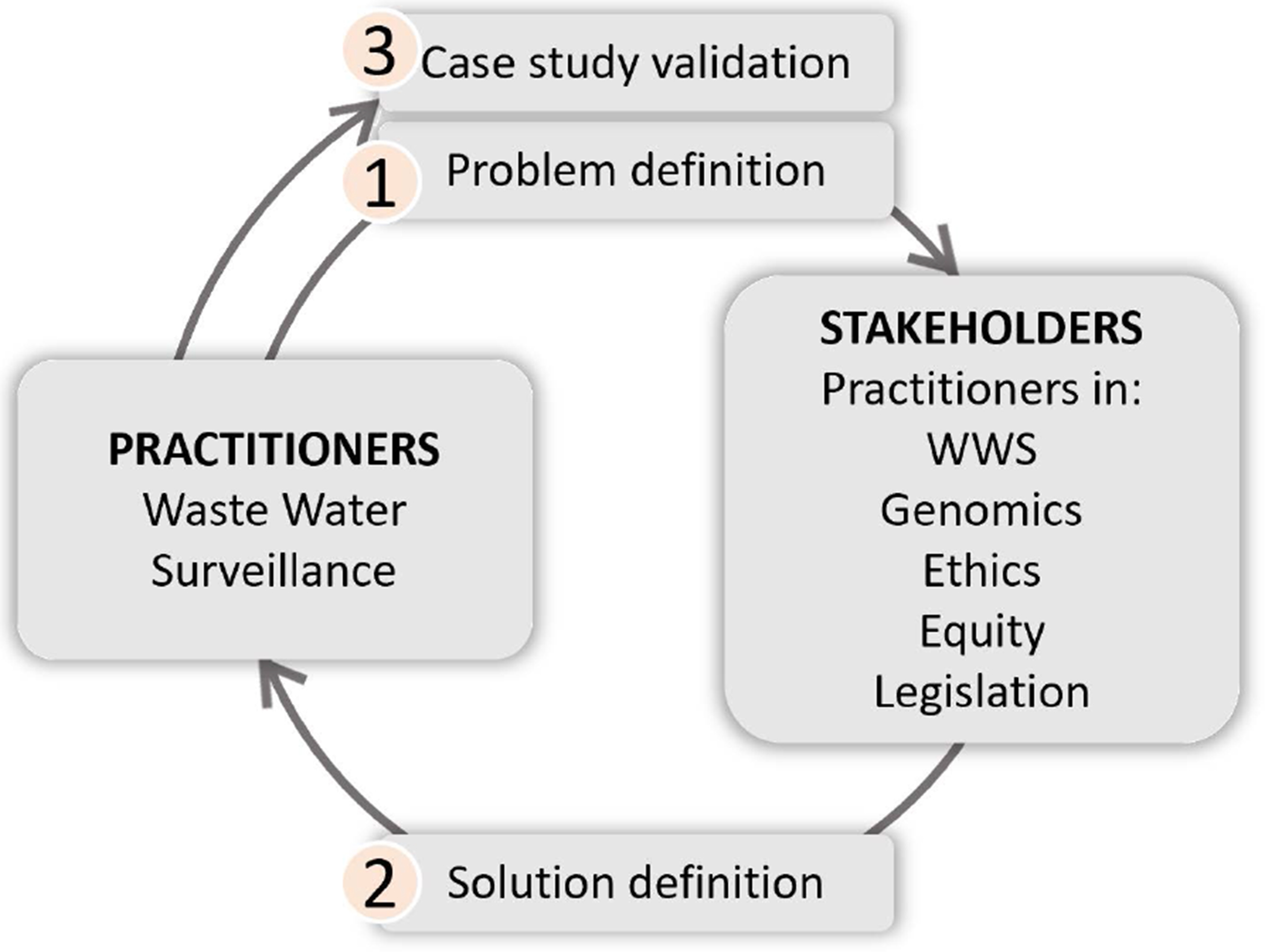
Process to define and populate the two-dimensional matrix. An iterative approach was use to define the problem, populate and validate the framework. https://doi.org/10.1371/journal.pwat.0000422.g001

The process was undertaken as a consultative, iterative process with members of the PHA4GE Consortium and other colleagues working in this field (Fig 1), including specialists in: WWS, pathogen genomics, data management, ethics for human research, equity, law and data governance. These experts brought their expertise and experience in specific areas together, ensuring cross-sectoral design and experience-informed population of the matrix, which remains mutable so that updates and changes can be easily incorporated in order to continually improve the advisory content that can be offered without altering the form or functioning of the app. We drew on a large body of existing publications and resources, such as legislation – for example National Health and Data Privacy Acts such as the Protection of Personal Information Act and Health Act in South Africa, The General Data Protection Regulation of the UK, National Health Acts, The Nagoya Protocol); and ethics guidelines such as the WHO Guidelines on Ethical Issues in Public Health Surveillance.

**Fig 2.**
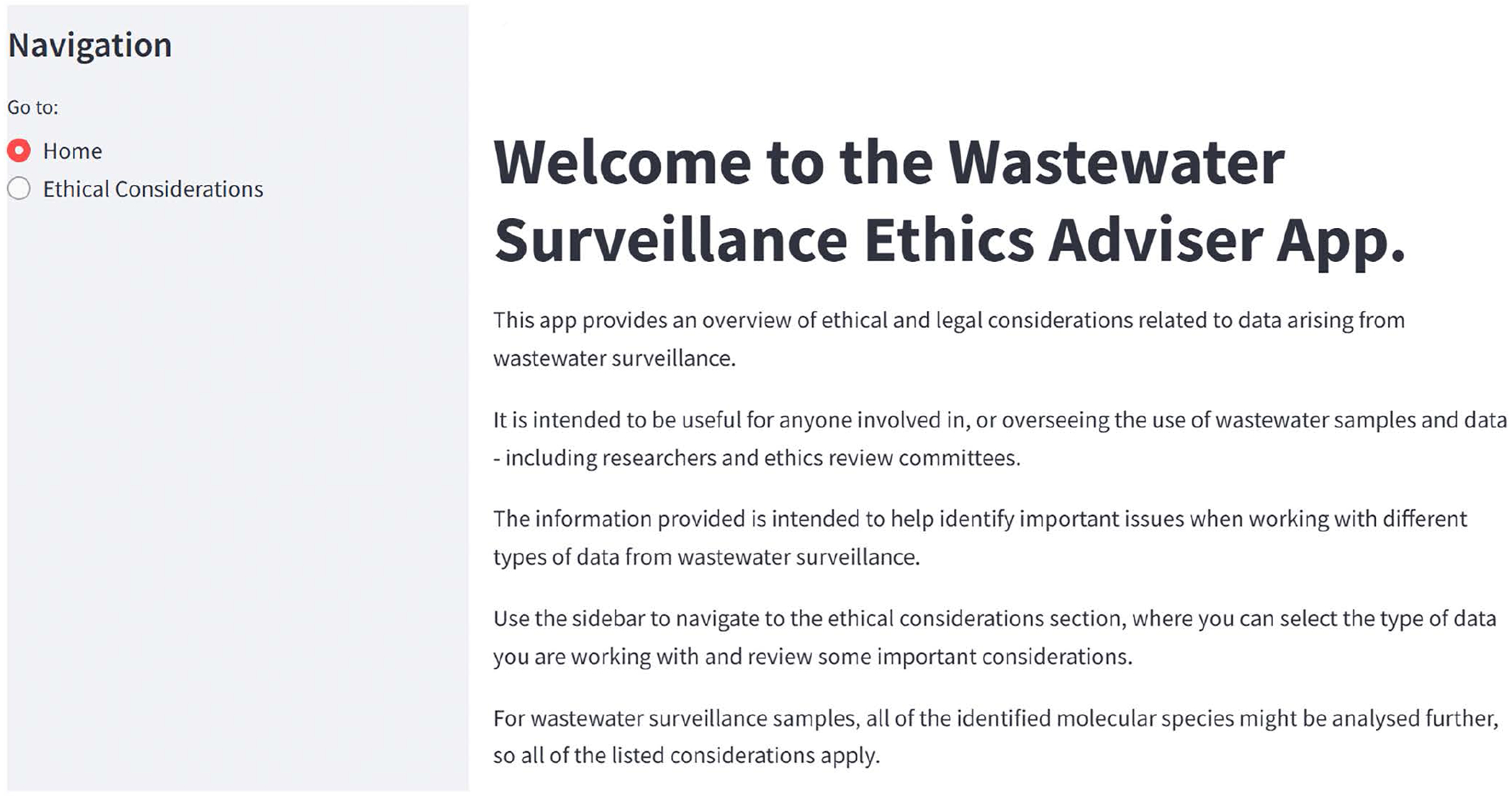
Home page for the WWS Ethics Adviser app, with introduction and instructions. https://doi.org/10.1371/journal.pwat.0000422.g002

**Fig 3.**
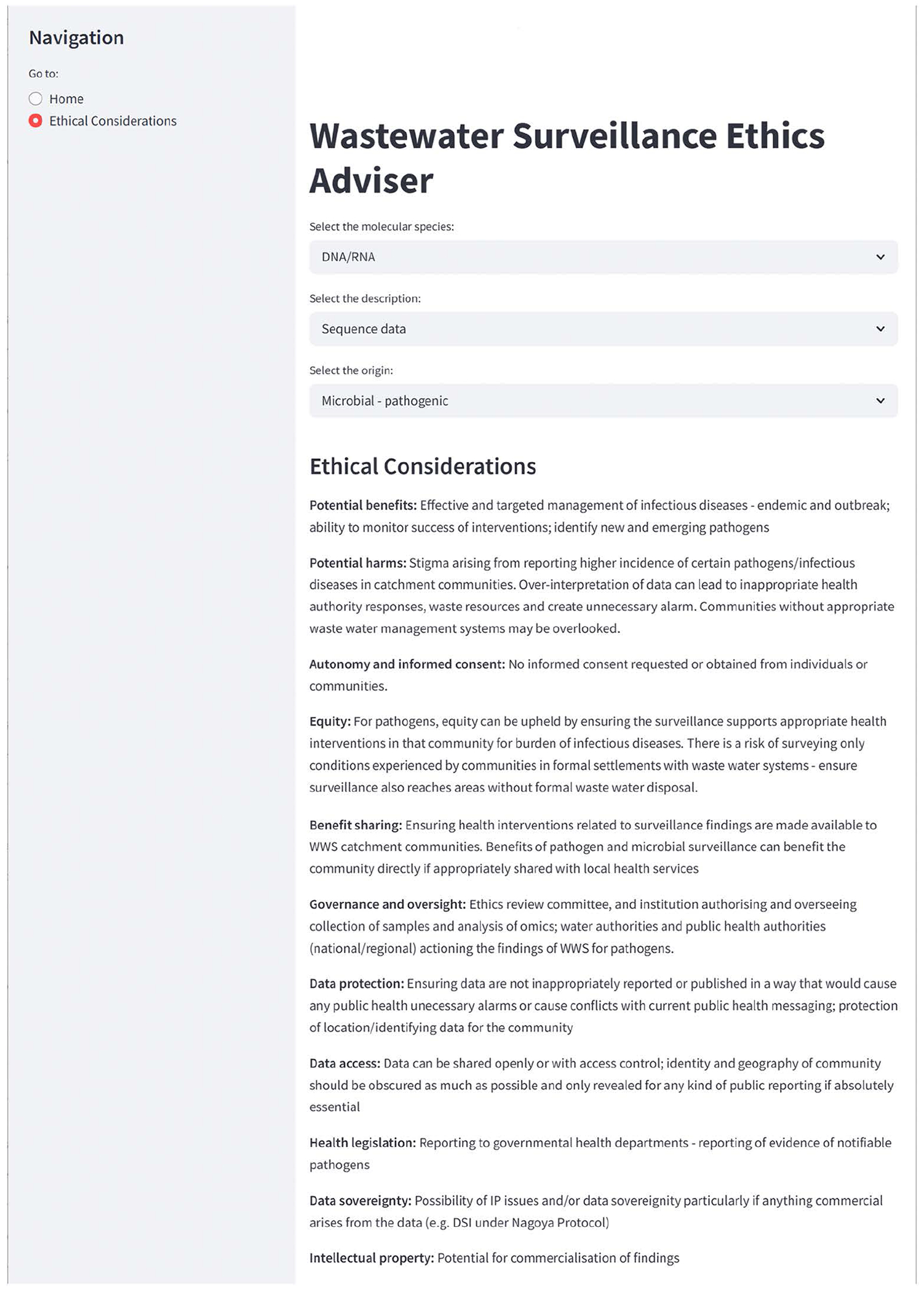
Selection page, with drop-down options for molecular types, subtypes and their origin, showing the relevant commentary with ethical, governance and legal alerts specific to the selected scenario. https://doi.org/10.1371/journal.pwat.0000422.g003

The matrix was used as the back-end content source for an online, browser-based, interactive application (Fig 2). To support exploration and decision-making around ethical considerations in WWS, we developed the interactive web-based tool using Streamlit (https://streamlit.io/), an open-source Python framework designed for the rapid development and deployment of data scripts. This application, called the WWS Ethics Adviser, first prompts the user to select the WWS molecular type and subtypes of particular interest to them, and their origin, then guides the user through a series of ethical and governance alerts relevant to that scenario, presenting tailored considerations and recommendations based on user input (Fig 3). All logic and interface components were written in Python, with deployment managed via Streamlit’s cloud platform using a GitHub repository for sourcefile storage.

We have applied the WWS Ethics Advisor App for user scenarios provided by key stakeholders to test utility of the provided advice. In addition, on the home page, a link is also provided for optional and voluntary feedback on the usability and content provided by the App, so that the user experience can be monitored on an ongoing basis and concerns from end users can be addressed as they are received.

This application is openly available online and can be accessed at https://wastewater-surveillance-ethics.streamlit.app/. As an online, browser-based tool, the app is openly available for global access.

## Results and discussion

We populated all cells with content where appropriate and kept the alerts as generalised and accessible as feasible. For some of the intersecting cells of the matrix there was no relevant or logical commentary or advice to be captured or some of the content was redundant across the cells, and these were captured as “not applicable” – for example, it would not be logical to attempt to offer ethical advice for the intersection of “autonomy and informed consent” with “Drugs – veterinary”, but it is appropriate to provide an alert for this molecule type regarding the potential “protection of commercially sensitive/patented information”. The two-dimensional framework underlying the online tool enables the user to select the fields that specifically describe the molecular type, subtype and source that they are working with, and the matched content consisting of relevant ethical, legal and governance alerts is selected from the underlying matrix and displayed. The user-friendly interface makes complex ethical, legal and governance information accessible to practitioners with very varied levels of expertise in these areas.

The content was restricted to short comments using non-technical language and is intended to provide generalised alerts about key issues to consider in each scenario, rather than providing detailed technical solutions for each possible data or sample subtype. Some examples of the types of issues that are flagged are provided in the Case Study (Text box), and are presented as a demonstration of the functionality and validation of output for a series of high priority use cases identified by our stakeholders, reflecting the different domains in which they operate (Fig 1). The WWS Ethics Adviser app is thus intended to prompt users about some of the key issues to be considered, without aiming to provide exhaustive or detailed recommendations for every possible use case, and this approach helps ensure the tool remains user-friendly, practical and adaptable. This also reflects an *important potential limitation* of the WWS Ethics Adviser, namely that it does not offer legal advice, but rather provides generalized alerts to possible ethics, equity and legislative considerations.

Additionally, although the WWS Ethics Adviser offers accessible, scenario-based guidance, it does not replace the need for users to apply their own professional judgement within the legal, ethical and governance requirements of their jurisdiction. Legislation may vary widely between countries, so users must ensure that any actions prompted by the tool are interpreted in light of local statutes, institutional policies and appropriate community protections. In this sense, the app is designed to prompt reflection rather than deliver definitive ethical or legal determinations, and acts as a decision support tool (similar to [22]) rather than aiming to replace the decision-maker. Recognising this distinction is essential to avoid overreliance or misinterpretation of the outputs, and to ensure accountability remains firmly with the decision-maker rather than being ceded to the WWS Ethics Advisor. Future work includes the dissemination of the app where it may be best used, for example to Ethics Review Committees, National Health Surveillance Laboratories, and WWS research practitioners; and engaging with users in these communities on an ongoing basis to understand usability of the WWS Ethics Advisor and its content.

This paper presents a practical, user-friendly tool to support researchers, ethics committees, and other stakeholders in navigating the complex ethical, legal, and equity considerations surrounding wastewater surveillance (WWS). The web-based application offers structured guidance based on key variables, including the type and source of molecular data, and is intended to assist those who may not have the time or capacity to engage deeply with the full breadth of relevant policy documents and principles but are nonetheless committed to ethical and lawful research practices.

While the tool provides scenario-based guidance for specific molecule types, users must remain aware of the additional responsibilities that arise from the generation and processing of complex biological data. Depending on the technologies employed— and particularly in the use of high-throughput methods such as next-generation sequencing—data arising from other molecules in the sample may be inadvertently captured. A common example of this is the unintended inclusion of human DNA when sequencing microbial communities. In such cases, appropriate bioinformatics pipelines must be used to identify and remove these data before analysis or onward data sharing.

The storage of WWS samples poses substantial additional ethical and legal challenges. By definition, a WWS sample contains all molecular types, most likely from most, if not all types of contributing sources, offering the potential to derive different types of highly sensitive data beyond the original purpose of collection. For example, samples collected for pathogen surveillance might later be requested by a governmental drug enforcement agency to support forensic investigations into drug use and supply within a population. Such scenarios raise complex ethical dilemmas regarding consent, community risk and competing legal obligations. Given the high potential for secondary use and repurposing, the ethical management of WWS samples therefore requires careful, intentional and case-specific governance.

For these reasons, the WWS Ethics Advisor app does not attempt to offer prescriptive guidance on sample storage and reuse. Rather, the matrix underpinning the tool encapsulates considerations that apply broadly and variably depending on the context of use. The tool is designed to complement institutional and researcher-initiated processes and encourage ethical reflection, while recognising the practical constraints under which many research teams and review bodies operate. In this way, the WWS Ethics Adviser serves not as a prescriptive authority, but as a practical prompt for reflective and responsible practice in a rapidly evolving field.

## Supplementary Material

Supplementary table 1: Matrix with first dimension of ethical and legal considerations and second dimension with molecular species found in waste water, showing content used to populate the online WWS Ethics Adviser **S1 Table. Content for the two-dimensional matrix underlying the browser-based ethics advisor application.** (PDF)

## Figures and Tables

**Table 1.** First dimension.

Molecular entities	Molecular subtype	Origin
DNA/RNA	Sequence data	Human Microbial - pathogenic Microbial - non-pathogenic Plant Animal
Protein	Sequence data	Human Microbial Plant Animal
Chemical	Metabolites	Human Microbial Plant Animal
Chemical	Drugs - medical Drugs - recreational	Human
Chemical	Drugs - veterinary	Animal
Chemical	Industrial	Environmental
Chemical	Agricultural	Environmental

https://doi.org/10.1371/journal.pwat.0000422.t001

**Table 2.** Second dimension.

Theme	Considerations
Ethics	Potential benefits Potential harms Autonomy and Respect Community engagement Informed consent
Justice	Equity Benefit sharing
Governance	Responsible party Data protections Access
Legislation	Health legislation Privacy Acts Environmental legislation Agricultural legislation Conservation legislation Biodiversity protocols and conventions Data sovereignty Intellectual property

https://doi.org/10.1371/journal.pwat.0000422.t002

## Data Availability

There are no data associated with this manuscript. The matrix containing the content for the app is provided as a supplementary file.
