# Supplementary material for: Accessible ethics and legal advice for wastewater surveillance: The WWS ethics adviser app
Source: PLOS Water. Author manuscript; Available in PMC 2026 May 13. (PMC13166137; doi:10.1371/journal.pwat.0000422)
Supplement: Supplementary table 1: Matrix with first dimension of ethical and legal considerations and second dimension with molecular species found in waste water, showing content used to populate the online WWS Ethics Adviser — S1 Table. Content for the two-dimensional matrix underlying the browser-based ethics advisor application. (PDF) [file NIHMS2163886-supplement-Supplementary_table_1__Matrix_with_first_dimension_of_ethical_and_legal_considerations_and_second_dimension_with_molecular_species_found_in_waste_water__showing_content_used_to_populate_the_online_WWS_Ethics_Adviser.pdf]

Supplementary table 1: Matrix with first dimension of ethical and legal considerations and second dimension with molecular species found in waste water, showing content used to populate the online WWS Ethics Adviser

Molecular species	Description	Origin	Potential benefits	Potential harms	Autonomy and informed consent	Equity	Benefit sharing	Governance	Data protections	Access	Legislation	Health legislation	Data sovereignty	IP
**DNA/RNA**	**Sequence data**	**Human**	Only contribution to general knowledge about population structure and ancestry; no clear benefits to community where DNA originates. Could possibly be used to estimate frequency of known disease alleles/Mendelian disease alleles in a community or population.	Community level loss of privacy, stigma; Personal loss of privacy. Some ancestry studies provide information in conflict with a community or populations historical and cultural beliefs; some ancestry studies may highlight certain population groups in a negative way	No informed consent has been requested or granted from individuals re: sequencing of their DNA; community engagement and community level consent/assent essential for any analysis of aggregate/metagenome human DNA	Ensure community engagement with local community teams who share language and cultural identity with the communities being engaged, avoid power differentials and inequitable representation of the community (e.g. control of the process by leaders with specific interests/conflict of interests)	Not clear that there are any benefits to be gained from Human DNA genome sequencing in this context, and not clear what benefits could be shared. Perhaps there is an opportunity for compensatory benefits relating to permissions to analyse human DNA/metagenomes in the sample, but	Ethics review committee, and institution authorising and overseeing collection of samples and analysis of DNA	Protection of identifying and highly personal data, and especially if there is no individual informed consent, or community level majority consent for use of metagenome data - highest level of protection and onward use oversight needed	Aggregate data, highly protected. Onward studies need oversight and ethical clearance due to risk of community harms; identity and geography of community should be obscured	Protection of personal information/data protection acts; right to privacy	Engage with the local health service for any community health information that is derived, to ensure compliance with health service requirements	NA	NA
**DNA/RNA**	**Sequence data**	**Microbial - pathogenic**	Effective and targeted management of infectious diseases - endemic and outbreak; ability to monitor success of interventions; identify new and emerging pathogens	Stigma arising from reporting higher incidence of certain pathogens/infectious diseases in catchment communities. Over-interpretation of data can lead to inappropriate health authority responses, waste resources and create	No informed consent requested or obtained from individuals or communities.	For pathogens, equity can be upheld by ensuring the surveillance supports appropriate health interventions in that community for burden of infectious diseases. There is a risk of surveying only conditions experienced by communities in formal settlements with waste water	Ensuring health interventions related to surveillance findings are made available to WWS catchment communities. Benefits of pathogen and microbial surveillance can benefit the community directly if appropriately shared with local health services	Ethics review committee, and institution authorising and overseeing collection of samples and analysis of omics; water authorities and public health authorities (national/regional) actioning the findings of WWS for pathogens.	Ensuring data are not inappropriately reported or published in a way that would cause any public health unecessary alarms or cause conflicts with current public health messaging; protection of location/identifying data for the community	Data can be shared openly or with access control; identity and geography of community should be obscured as much as possible and only revealed for any kind of public reporting if absolutely essential	NA	Reporting to governmental health departments - reporting of evidence of notifiable pathogens	Possibility of IP issues and/or data sovereignty particularly if anything commercial arises from the data (e.g. DSI under Nagoya Protocol)	Potential for commercialisation of findings
**DNA/RNA**	**Sequence data**	**Microbial - nonpathogenic**	Identification of species with biotechnological applications; ecological evaluation of ecosystem. Possibility of research benefits, and the potential for	Unmanaged/unregulated exploitation of natural resources	Communities are custodians of their local natural resources and should be stakeholders in any new findings	Community involvement in the research and follow-on development arising from microbial sequence data taken from their wastewater	For microbial data used for commercial purposes, benefits of commercialisation should be shared with communities. Potential for sharing benefits from	Institutional tech transfer offices and lawyers represent the institute as the responsible party.	IP protection wrt new commercial developments arising from microbial data	Governed by IP and patent law. (Potentially also Nagoya Protocol DSI amendments)	NA	NA	Possibility of IP issues and/or data sovereignty particularly if anything commercial arises from the data (e.g. DSI under Nagoya Protocol)	Potential for commercialisation of findings
**DNA/RNA**	**Sequence data**	**Plant**	Potential benefits in environmental exploration/preservation (new species information)? Potential for commercialisation	Unmanaged/unregulated exploitation of natural resources and/or endangered species	Communities are custodians of their local natural resources and should be stakeholders in any new findings	Community involvement in the research and follow-on development arising from plant data taken from their wastewater; local involvement in custodianship and management	Benefits of commercialisation; also custodianship and shared management of local plant resources. Sharing knowledge and custodianship of new or rare species	Institutional tech transfer offices and lawyers represent the institute as the responsible party.	IP protection wrt new commercial developments arising from plant data, actions to protect rare/endangered species from being located or inappropriately exploited	Governed by IP and patent law. (Potentially also Nagoya Protocol DSI amendments)	Legislation on conservation and protection of the natural environment	NA	Possibility of IP issues and/or data sovereignty particularly if anything commercial arises from the data (e.g. DSI under Nagoya Protocol). Possibility of IP and patent issues around plant strains	Potential for commercialisation of findings
**DNA/RNA**	**Sequence data**	**Animal**	Potential benefits in environmental exploration/preservation (new species information, surveillance of animal populations)?	Unmanaged/unregulated exploitation of natural resources and/or endangered species	Communities are custodians of their local natural resources and should be stakeholders in any new findings	Community involvement in the research and follow-on development arising from animal data taken from their wastewater; local involvement in	Custodianship and shared management of local animal resources, sharing knowledge and custodianship of new or rare species	Institutional tech transfer offices and lawyers represent the institute as the responsible party.	IP protection wrt new commercial developments arising from animal data, actions to protect rare/endangered species from being located or	Governed by IP and patent law. (Potentially also Nagoya Protocol DSI amendments)	Legislation on conservation and protection of the natural environment	NA	Possibility of IP issues and/or data sovereignty particularly if anything commercial arises from the data (e.g. DSI under Nagoya Protocol). Possibility of IP	NA
**Protein**	**Sequence data**	**Human**	Understanding prevalence of some common diseases in the population;	Community-level reporting may lead to stigma for communities	Community discussion of analyses	Involvement of local individuals in analysis/interpretation	Ensuring appropriate interventions based on findings, and ensuring they benefit	Ethics review committee, and institution authorising and overseeing collection of	Protecting community identity	No implications	No implications	No implications	NA	NA
**Protein**	**Sequence data**	**Microbial**	Potential for commercialisable products	No specific harms	Communities are custodians of their local natural resources and should be stakeholders in any new findings	Involvement of local individuals in analysis/interpretation	If microbial findings are used for commercial purposes, benefits of commercialisation should be shared with communities. Any pathogen-related	Institutional tech transfer offices and lawyers represent the institute as the responsible party.	IP protection wrt to new commerical developments arising from microbial data	Governed by IP and patent law. (Potentially also Nagoya Protocol DSI amendments)	NA	Reporting of evidence of notifiable pathogens	Possibility of IP issues and/or data sovereignty particularly if anything commercial arises from the data (e.g. DSI under Nagoya Protocol). Possibility of	Potential for commercialisation of findings
**Protein**	**Sequence data**	**Plant**	Potential for commercialisable products	No specific harms	Communities are custodians of their local natural resources and should be stakeholders in any new findings	Involvement of local individuals in analysis/interpretation	Benefits of commercialisation; also custodianship and shared management of local plant resources. Findings should benefit the local community	Institutional tech transfer offices and lawyers represent the institute as the responsible party.	IP protection wrt new commercial developments arising from plant data, actions to protect rare/endangered species from being located or	Governed by IP and patent law. (Potentially also Nagoya Protocol DSI amendments)	NA	NA	Possibility of IP issues and/or data sovereignty particularly if anything commercial arises from the data (e.g. DSI under Nagoya Protocol). Possibility of IP	Potential for commercialisation of findings
**Protein**	**Sequence data**	**Animal**	Understanding animal population health and diet	No specific harms	Communities are custodians of their local natural resources and should be stakeholders in any new findings	Involvement of local individuals in analysis/interpretation	Benefits of commercialisation; also custodianship and shared management of local animal resources. Findings should benefit the local community	Institutional tech transfer offices and lawyers represent the institute as the responsible party.	IP protection wrt new commercial developments arising from animal data, actions to protect rare/endangered species from being located or	Governed by IP and patent law. (Potentially also Nagoya Protocol DSI amendments)	NA	NA	Possibility of IP issues and/or data sovereignty particularly if anything commercial arises from the data (e.g. DSI under Nagoya Protocol). Possibility of IP	Potential for commercialisation of findings
**Chemical**	**Metabolites**	**Human**	Understanding prevalence of some common diseases in the population; understanding diet in the population (e.g. caffeine and alcohol metabolites)	Concerns for stigma for communities	Community consent may be needed for this kind of surveillance	Must ensure that communities surveyed receive interventions depending on their needs as determined by the research	Ensure communities receive appropriate interventions based on findings	Ethics review committee, and institution authorising and overseeing collection of samples and analysis	Protection of community identity	No implications	No implications	NA	NA	NA
**Chemical**	**Metabolites**	**Microbial**	Potential for commercialisable products; understand shifts in microbial populations that reflect factors like climate change or pollution	No specific harms	Include and provide information to community during process	Involvement of local individuals in analysis/interpretation	If microbial findings are used for commercial purposes, benefits of commercialisation should be shared with communities. Any pathogen-related findings should be used to improve health/reduce risk for community	Institutional tech transfer offices and lawyers represent the institute as the responsible party.	IP protection wrt new commercial developments arising from microbial data	Governed by IP and patent law.	NA	Reporting of evidence of notifiable pathogens	Possibility of IP issues and/or data sovereignty particularly if anything commercial arises from the data . Possibility of IP and patent issues due to potential for commercialisation of findings	
**Chemical**	**Metabolites**	**Plant**	Potential for commercialisable products; understand shifts in plant metabolism that reflects factors like climate change or pollution	No specific harms	Include and provide information to community during process	Involvement of local individuals in analysis/interpretation	Benefits of commercialisation; also custodianship and shared management of local plant resource. Findings should benefit the community.	Institutional tech transfer offices and lawyers represent the institute as the responsible party.	IP protection wrt new commercial developments arising from plant data, actions to protect rare/endangered species from being located or inappropriately exploited	Governed by IP and patent law.	NA	NA	Possibility of IP issues and/or data sovereignty particularly if anything commercial arises from the data . Possibility of patent issues .	Potential for commercialisation of findings
**Chemical**	**Metabolites**	**Animal**	Understanding animal population health and diet	No specific harms	Include and provide information to community during process	Involvement of local individuals in analysis/interpretation	Benefits of commercialisation; also custodianship and shared management of local animal resources, findings should benefit the community	Institutional tech transfer offices and lawyers represent the institute as the responsible party.	IP protection wrt new commercial developments arising from animal data, actions to protect rare/endangered species from being located or inappropriately exploited	Governed by IP and patent law.	NA	NA	Possibility of IP issues and/or data sovereignty particularly if anything commercial arises from the data. Possibility of patent issues.	Potential for commercialisation of findings
**Chemical**	**drugs - medical**	**Human**	Assessment of AMR risk (antibiotics use) to inform interventions; assessment of drugs with a risk of misuse (e.g. opioids)	Stigma for communities based on findings	NA	Local community members involved in interpretation of findings	Share relevant information with local health authorities, ensure information is used to improve the lives of communities	Ethics review committee, and institution authorising and overseeing collection of samples and analysis	Protection of community identity	Governed by IP and patent law	NA	NA	NA	Patent law around composition of some medications
**Chemical**	**drugs - recreational**	**Human**	Assessment of recreational drug use to inform interventions, identify sites of illegal drug production and/or wholesale storage	Stigma for communities based on findings	NA	Local community members involved in interpretation of findings	Alerts informing interventions for illegal drug production and use, information to support local police and Drug Enforcement Agencies; information should be used to improve the lives of community members	Ethics review committee, and institution authorising and overseeing collection of samples and analysis	Protection of community identity	Governed by Drug Enforcement and criminal law	Legislation on illegal substances, reporting to drug enforcement agencies	NA	NA	NA
**Chemical**	**drugs - veterinary**	**Animal**	Assessment of OneHealth impact, AMR, domestic animal processing practices, to detect health risks and AMR outbreaks	Stigma for communities based on findings; potential impact on food production sector	Not Applicable	Local community members involved in interpretation of findings	Alerts informing interventions for environmental level of veterinary drugs (local health risks, other risks such as AMR development), public warnings and information where appropriate, ensuring findings benefit the community	Institutional tech transfer offices and lawyers represent the institute as the responsible party.	Protection of commercially sensitive/patented information	Governed by IP and patent law	Agricultural legislation, reporting to Agricultural agencies	NA	NA	Patent law around composition of some medications
**Chemical**	**Industrial**	**Environmental**	Profile pollution risks to population	No specific harms	Not Applicable	Local community members involved in interpretation of findings	Ensuring findings are used to improve environment of local communities: All populations receive benefits of decreased levels of pollutants and contaminants, and evidenced-based clean up of the environment	Institutional tech transfer offices and lawyers represent the institute as the responsible party.	Protection of commercially sensitive/patented information	Governed by IP and patent law	Compliance with environmental protection and pollution legislation	NA	NA	Patent law around composition of chemicals
**Chemical**	**Agricultural**	**Environmental**	Assessment of pesticides, herbicides and other, to identify risks to populations and the ecosystem.	No specific harms	Not Applicable	Local community members involved in interpretation of findings	Alerts for pollution levels to inform interventions and provide local warnings and guidance where relevant; All populations receive benefits of decreased levels of pollutants and contaminants, and evidenced-based clean up of the environment	Institutional tech transfer offices and lawyers represent the institute as the responsible party.	Protection of commercially sensitive/patented information	Governed by IP and patent law	Agricultural legislation, reporting to Agricultural agencies, as well as environmental protection/pollution agencies	NA	NA	NA
